# Friction Stir Processing of Copper-Coated SiC Particulate-Reinforced Aluminum Matrix Composite

**DOI:** 10.3390/ma11040599

**Published:** 2018-04-13

**Authors:** Chih-Wei Huang, Jong-Ning Aoh

**Affiliations:** Advanced Institute of Manufacturing with High-Tech Innovations (AIM-HI), Department of Mechanical Engineering, National Chung-Cheng University, Chia-Yi 62102, Taiwan; red740629@hotmail.com

**Keywords:** friction stir processing (FSP), aluminum matrix composites (AMCs), core-shell SiC/Cu particulate reinforcement, intermetallic compounds (IMCs), dispersion strengthening mechanisms

## Abstract

In the present work, we proposed a novel friction stir processing (FSP) to produce a locally reinforced aluminum matrix composite (AMC) by stirring copper-coated SiC particulate reinforcement into Al6061 alloy matrix. Electroless-plating process was applied to deposit the copper surface coating on the SiC particulate reinforcement for the purpose of improving the interfacial adhesion between SiC particles and Al matrix. The core-shell SiC structure provides a layer for the atomic diffusion between aluminum and copper to enhance the cohesion between reinforcing particles and matrix on one hand, the dispersion of fine copper in the Al matrix during FSP provides further dispersive strengthening and solid solution strengthening, on the other hand. Hardness distribution and tensile results across the stir zone validated the novel concept in improving the mechanical properties of AMC that was realized via FSP. Optical microscope (OM) and Transmission Electron Microscopy (TEM) investigations were conducted to investigate the microstructure. Energy dispersive spectrometer (EDS), electron probe micro-analyzer (EPMA), and X-ray diffraction (XRD) were explored to analyze the atomic inter-diffusion and the formation of intermetallic at interface. The possible strengthening mechanisms of the AMC containing Cu-coated SiC particulate reinforcement were interpreted. The concept of strengthening developed in this work may open a new way of fabricating of particulate reinforced metal matrix composites.

## 1. Introduction

Aluminum alloys are extensively used for structural applications in aerospace, military, and transportation industries due to their low density, high specific strength, and good corrosion resistance. However, their poor wear resistance has limited their tribological applications. In comparison to aluminum alloys, aluminum matrix composites (AMCs) that were reinforced with discontinuous reinforcements, such as Al_2_O_3_, TiB_2_, or SiC ceramic particles exhibit higher mechanical strength and superior tribological properties [1,2,3,4]. The performance and characteristics of AMCs depend on several aspects, such as uniformity of reinforcement particles distribution and the interfacial coherence between reinforcement particles and aluminum matrix [5]. However, clustering, porosity, and the agglomeration of particulate reinforcement in the matrix are major concerns in the fabrication of AMCs using conventional melting processes, such as stir casting, liquid metal infiltration, or powder metallurgy [6,7,8,9,10]. Friction stir processing (FSP) that was proposed by Mishra et al. [5] opens a new way for the fabrication of AMCs. Friction stir processing (FSP) is developed based on Friction stir welding invented by The Welding Institute (TWI) in 1991. The key parameters of the process are tool geometry, rotating speed, and traverse speed, which have significant effects on the microstructure and mechanical properties [11,12]. During the process, a non-consumable rotating tool with a shoulder and pin is plunged into the material and move along the welding direction. Because of the heat generation by frictional heat and stirring, the material is softened without melting. Due to the plastic deformation, the reinforcing particles are mixed into matrix [13]. A localized particulate reinforced aluminum matrix composites is formed in the stir zone. This process could overcome agglomeration of particles and achieves a uniform distribution of particulate reinforcement [14,15]. However, the tool life is a major concern for fabricating AMCs, some research applied laser-assisted friction stir welding (LAFSW) to join the aluminum plates. Because of the laser pre-heat, tool wear decreased and a higher traverse speed was obtained by the application of LAFSW [16]. During friction stir process, the hard and brittle particulate reinforcements were mixed into the ductile aluminum substrate. The bonding interface between ceramic particles and the metal matrix are incoherent. The interfacial characteristics, including the interfacial bonding structure, the formation of intermediate phase, and the difference in thermal expansion are complicated when compared with those AMCs containing metallic reinforcements. The complexity of interface reactions affects the bonding strength between ceramic particles and aluminum matrix, which would further affect the mechanical properties of the AMCs.

In order to modify the surface characteristics of the reinforced particles, we use the electroless plating technique to coat copper film on SiC particles. The core-shell structure SiC/Cu was designed to improve the interfacial adhesion between SiC particles and aluminum matrix. In addition, the copper coating provides an inter-diffusion layer between aluminum matrix and copper coating to achieve better cohesion between reinforcing particles and matrix [17,18]. Furthermore, a part of the copper film may be dispersed in the matrix during friction stir process and then also provide an additional effect of solid solution strengthening. Optical microscope (OM), Transmission Electron Microscopy (TEM), high resolution transmission electron microscopy (HRTEM), energy dispersive spectrometer (EDS), X-ray diffraction (XRD), and electron probe micro-analyzer (EPMA) analyses were conducted to investigate the microstructure evolution and to interpret the possible strengthening mechanisms that were contributed by the addition of the copper-coated SiC particles into Al matrix via FSP.

## 2. Materials and Methods 

**Figure 1:**
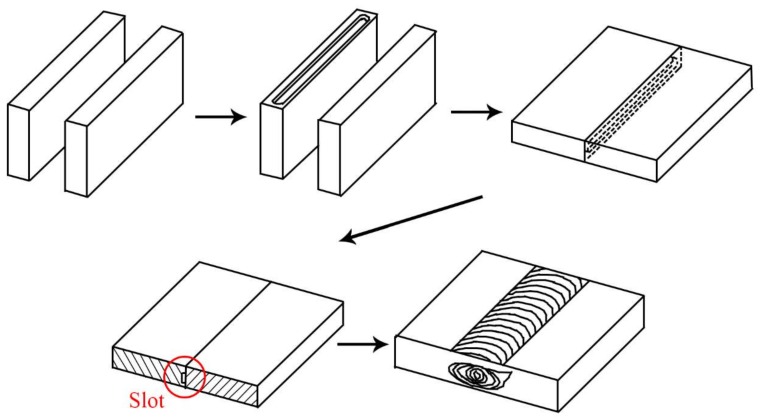
Schematic illustration on stirring reinforced particles into Al6061 stir zone by friction stir processing (FSP).

**Figure 2:**
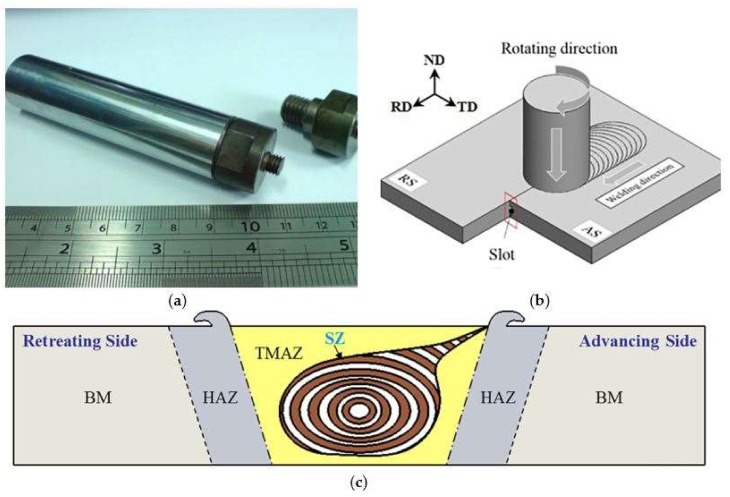
(**a**) Interchangeable FSP tool, (**b**) Schematic illustration of FSP, and (**c**) Definition of the friction stir welding zone.

In this study, as-rolled aluminum Al6061-T651 plates with dimensions of 240 × 100 × 6 mm were chosen. The chemical composition and mechanical properties of this material are listed in Table 1 and Table 2. As shown in Figure 1, a slot was prepared on one butt end of an Al6061 base plate for inserting the reinforcement particles into the matrix prior to FSP. The FSP tools was made of SKD61 mold steel with a shoulder of 20 mm in diameter and a left threaded pin (pitch 1 mm) of 6 mm in diameter and 5 mm in length. After a series of parameter study, the tool rotating speed of 1000 rpm clockwise and traverse speed of 1.2 mm/s were chosen. The tool tilt angle (angle between spindle and work piece normal) of 2° was applied. The interchangeable of the FSP tool is shown in Figure 2a. The schematic illustration on how the copper-coated SiC particles were stirred into Al6061-T651 matrix is shown in Figure 2b. The directions of the plates, the slot at the butt joint, the rotating direction, the advancing side (AS), and the retreating side (RS) are marked. The definition of friction stir welding zone are shown in Figure 2c. There are four different zones in the transverse cross-section. Base metal (BM) is not affected by the process. The materials in Heat affected zone (HAZ) have not plasticity deformed but the properties of metallography have changed due to heat generation. Thermal-mechanical affected zone (TMAZ) is a non-recrystallization zone, but the grain have elongated plasticity due to the plastic deformation by stirring and the thermal effect of the friction heat. Stir zone (SZ) in which the material has been stirred by tool and the new equiaxed grains have formed with recrystallization.

**Figure 3:**
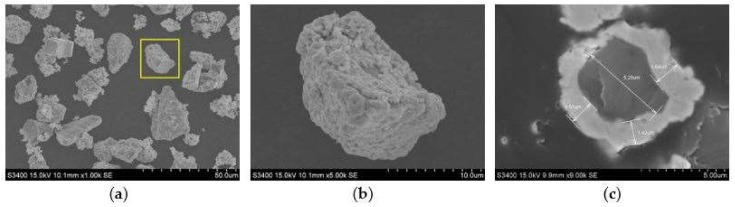
SEM micrographs of Cu-coated SiC particles, (**a**) Cu-coated SiC, and (**b**) magnification of Cu-coated SiC, (**c**) cross-sectional view.

SiC particles of nominal mesh size of 3–6 µm were chosen as the particulate reinforcements to be stirred into the Al6061 matrix. SEM micrographs of the Cu-coated SiC particles are shown in Figure 3. The surface morphology of the SiC particles that are shown in Figure 3a,b depicts a uniform and dense Cu-coated layer that is adhered to the particles. The thickness of the Cu coated layer is approximately 1.3–1.8 µm. A transverse section of the Cu-coated SiC particle shown in Figure 3c reveals uniform thickness and sound adhesion. The process details for electroless coating of Cu film on SiC particles are describes as follows:

Surface treatment for SiC particles is required before electroless plating. First, SiC particles were immersed in NaOH (aq) and then in HCl (aq) for 15 min for surface roughening, followed by sensitization in a stannous chloride (SnCl_2_·H_2_O) and hydrochloric acid (HCl) solution for 30 min. The SiC particles were then activated in palladium chloride (PdCl_2_) and hydrochloric acid for another 30 min. Solution of copper ion was prepared by dissolving CuSO_4_·5H_2_O in distilled water. The pH values of the solutions were adjusted by sodium hydroxide solution to 12.5 and the solution was heated to 60 °C. Ethylenediaminetetraacetic acid (EDTA) and 2,2′-bipyridyl was chosen as a complex agent, potassium ferrocyanide as stabilizer, and formaldehyde (35%) as reducing agent. The compositions of the solutions used in electroless plating process are listed in Table 3. The reaction time was 40 min. The Cu-coated SiC particles was then rinsed with distilled water and were dried at 70 °C for 15 h.

**Figure 4:**
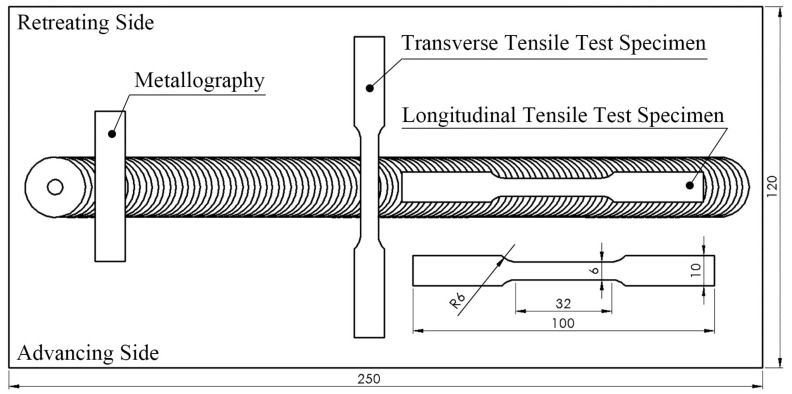
Schematic illustration of the specimen preparation from the friction stir processed zone.

Specimens for the optical microstructural observation on the stir zone and the thermo-mechanically affected zone (TMAZ) of the locally particulate-reinforced Al6061 AMC were prepared by grinding from No. 120 to No. 4000, polishing and then etching. The compositions of the etching solution are listed in Table 4. Investigation on the formation of intermetallic compounds in the stir zone was conducted using SEM (Hitachi S-3500N, Hitachi, Tokyo, Japan). XRD (BRUKER AXS-D2 PHASER, Cu Kα, 30 kV, 10 mA, BRUKER, Billerica, MA, USA), and EDS (OXFORD 6650, Hitachi, Tokyo, Japan), EPMA (JEOL JXA-8500F, JEOL, Tokyo, Japan), and TEM (JEM-2100F, JEOL, Tokyo, Japan) to identify the phases. The microhardness was measured using a micro-Vickers hardness tester (MHT-2) with a load of 200 g for 15 s on the samples cross section at 0.5 mm below friction stir processed surface. Mechanical properties of the locally particulate-reinforced Al6061 MMC specimens that were machined from the stirred zone (SZ) were carried out on an MTS 647 universal testing machine with an initial strain rate of 1 × 10^−3^ s^−1^. Figure 4 illustrates the schematic specimen preparation from the friction stir processed zone. The dimensions of the tensile specimens were determined according to ASTM E8-08 standard [19], 6 mm (length), 4 mm (width), and 20 mm in gauge length. Both longitudinal and transverse tensile specimens were prepared. For each condition, three specimens along each direction were tested.

## 3. Results

### 3.1. Microstructure and XRD Analysis

**Figure 5:**
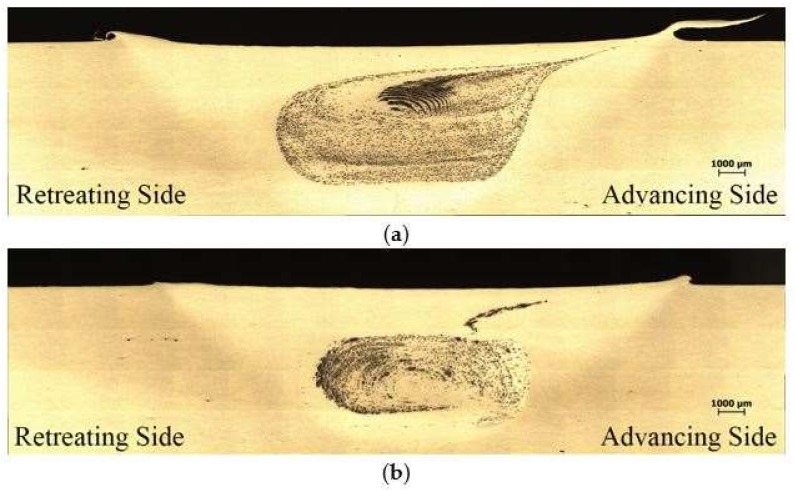
Cross-sectional macrographs of the locally particulate-reinforced Al6061 alloy fabricated by FSP. (**a**) Stir zone containing SiC particles, (**b**) stir zone containing Cu-coated SiC particles.

**Figure 6:**
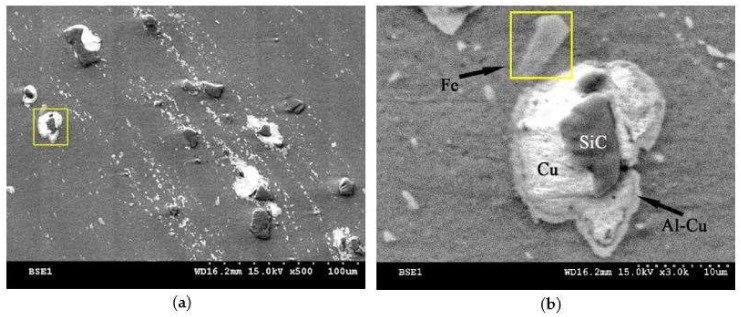
SEM micrographs of stir zone containing Cu-coated SiC particles. (**a**) Cu debries and SiC particles distribution along onion ring pattern, (**b**) magnification of (**a**) showing cross section of a Cu-coated SiC particle.

In general, the fabrication of locally particulate reinforced AMC has been realized in the stir zone by friction stir processing of bare SiC particles as well as Cu-coated SiC particles into Al6061 matrix. The stir zone that containing Cu-coated particles exhibited a different flow behavior as that containing the bare SiC particles. The parameter range to achieve a defect free stir zone for Cu-coated SiC particles was narrower than that of bare SiC. The shape and the flow pattern of the SZ containing bare SiC particles that are shown in Figure 5a reveals a larger SZ with very uniform particle distribution and a pronounced onion ring pattern in the upper part of the SZ. The stir zone containing Cu-coated SiC particles shown in Figure 5b reveals a slight smaller area containing reinforced particles and with less pronounced onion rings and flow arm with a few particles agglomeration along the flow arm. The lack of Cu-coated SiC particles in the upper 2 mm region in the SZ may be attributed to a less effective stir that is provided near the probe root adjacent to the shoulder. During FSP, some Cu-coating film was peeled off the SiC particles and formed scattered traces of extremely fine Cu debris coexisted with SiC or Cu-coated SiC particles which rendered macroscopic onion ring pattern visible as illustrated in Figure 6a. The magnification in Figure 6b shows the cross section of a Cu-coated SiC particle in the SZ after FSP. The SiC reinforcement particle is surrounded by a copper layer followed by a thin layer having different contrast from the bright copper layer that could be a mixture or compounds between Al and Cu. This was interpreted using EPMA analysis. The Cu-coated SiC particles exhibit a sound bonding with the Al substrate. A few tool debris was also found in the stir zone, which was then identified by EDS.

**Figure 7:**
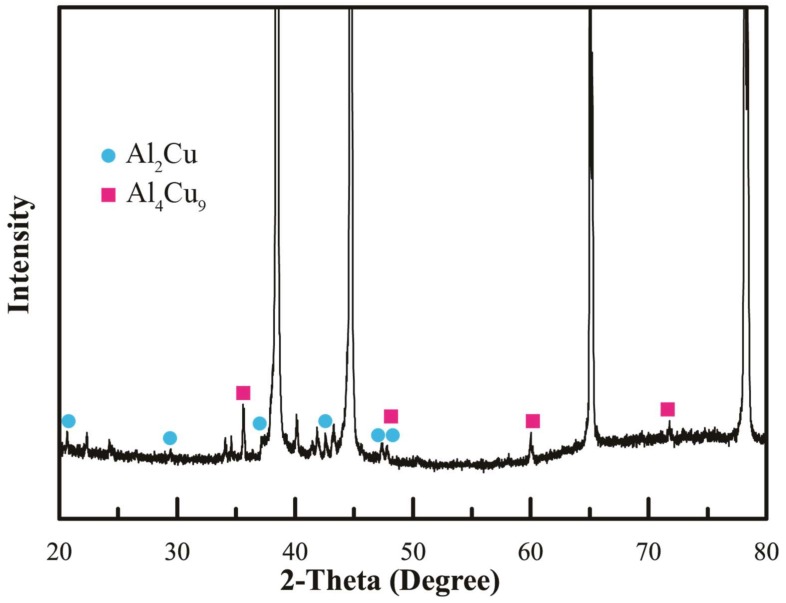
X-ray diffraction pattern of the friction stir zone containing SiC/Cu reinforcement.

According to the Al-Cu equilibrium phase diagram, several Al-Cu intermetallic compounds, including Al_2_Cu (θ), AlCu (η_2_), Al_3_Cu_4_ (ζ_2_), and Al_4_Cu_9_ (γ_1_), may form in the SZ. XRD analysis on stir zone containing Cu-coated SiC particles reinforcement in Figure 7 depicts the formation of two IMCs, Al_2_Cu, and Al_4_Cu_9_ after FSP. No other IMCs were found in XRD Heat that was generated both from severe plastic deformation and friction between particulate reinforcement and matrix may promote the atomic inter-diffusion between Cu layer and Al matrix and results in the formation of an Al-Cu IMC layer adjacent to the Cu layer. The formation of different Al-Cu IMCs depends on the in-situ reactions between the diffusion couple Al matrix/Al-Cu IMC/Cu layer [20,21]. The formation of IMC layer consisting of Al_2_Cu and Al_4_Cu_9_ could enhance the strengthening effect in addition to the particulate reinforcement in the SZ [22].

### 3.2. EPMA Analysis on Cu-Coated SiC Reinforcement in SZ

**Figure 8:**
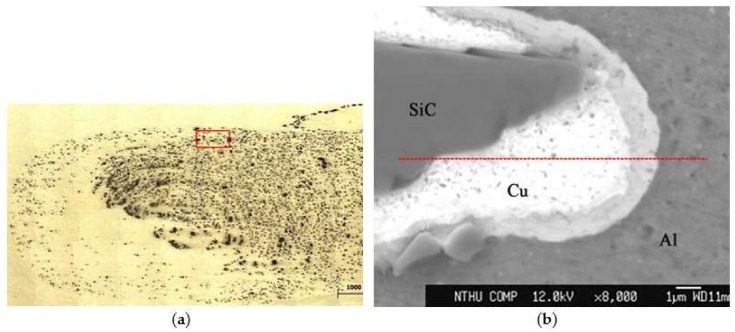
Micrographs showing the location of the electron probe micro-analyzer (EPMA) line scan across the Cu-coated SiC reinforced particle embedded in the matrix. (**a**) Lower magnification in optical microscope (OM), (**b**) higher magnification in SEM.

**Figure 9:**
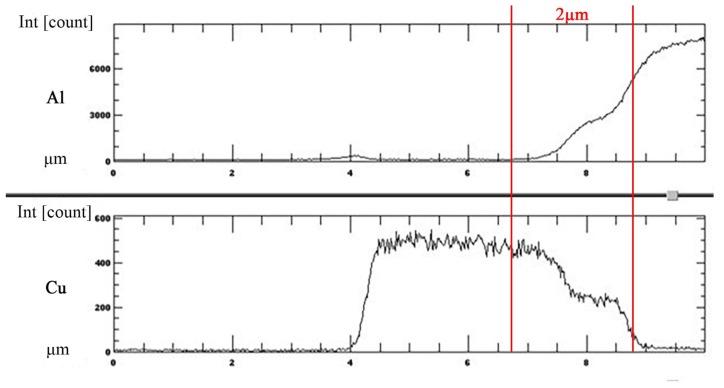
Al-SiC/Cu reinforcement with EPMA line scan across Cu-coated SiC and Al matrix showing Al and Cu distribution.

EPMA analysis was employed on the transverse section of a Cu-coated SiC particle that was embedded in the Al matrix to investigate the inter-diffusion between the copper coating and Al substrate. The EPMA line scan was conducted on the upper part of the SZ, as shown in Figure 8a. Detailed trace of line scan and points of interests are illustrated in Figure 8b. It is noted that a SiC particle is surrounded by a layer of copper deposit that was embedded in the Al matrix. An intermediate layer between the Cu deposit and Al matrix, which reveals that slightly different contrast can be observed. Figure 9 depicts the EPMA line scan result, showing the distribution of element Al and Cu along the scanned trace. Within a narrow range of 2 μm across the Al/Cu interface, it shows an increase in Al intensity and a decrease in Cu intensity. There is a cross-over of Al and Cu lines, which implies atomic inter-diffusion between Al and Cu. When combined with the XRD pattern in Figure 7, it can be inferred that the intermediate layer between the Cu coating and Al matrix that is shown in Figure 6b as well as in Figure 8b was formed due to the inter-diffusion of Al and Cu, and may contain mixture of Al/Cu and IMCs, such as Al_2_Cu and Al_4_Cu_9_. The thickness and the location of this inter-diffusion layer that was observed in micrographs agree very well with the EPMA result. It is reasonable to postulate that this inter-diffusion layer between the aluminum matrix and the coated Cu coating on SiC particles provides a metallurgical cohesion, which would strengthen the bond between the reinforced particles and the matrix and thus further enhance the effect of particulate reinforcement. In addition, a Wavelength-dispersive spectroscopy (WDS) analysis was conducted on the microstructure of Al matrix in SZ where neither Cu-coated SiC particles nor fine traces of Cu debris were found. The results revealed an elevated Cu content of 1.087 wt % when comparing with that of 0.284 wt % in the as-received base metal. The elevated Cu content may further enhance the effect of solid solution strengthening. 

### 3.3. HRTEM Investigation on Interface between SiC Reinforcement and Al Matrix

**Figure 10:**
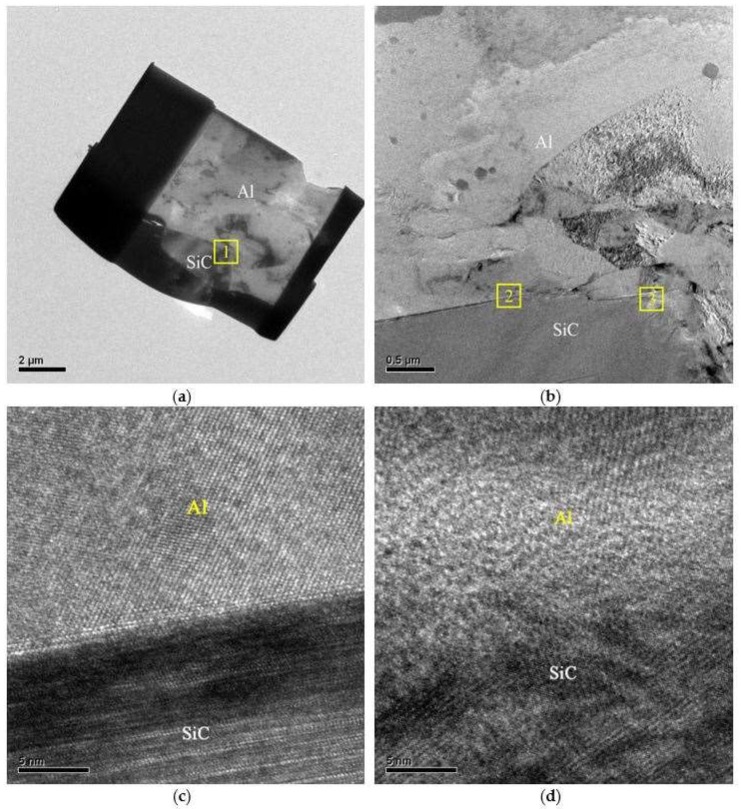
High resolution transmission electron microscopy (HRTEM) micrographs of stir zone containing bare SiC reinforcement. (**a**) Focus iron beam sample, (**b**) magnification of region 1 in (**a**), (**c**) magnification of region 2 in (**b**), (**d**) magnification of region 3 in (**b**).

Figure 10a shows a HRTEM specimen that was prepared by Focused ion beam (FIB) from SZ containing a bare SiC reinforced particle that was embedded in the aluminum matrix. Higher magnification of the region 1 in Figure 10a is depicted in Figure 10b. A sound cohesion between SiC particle and Al-matrix is clearly observed. HRTEM micrographs in Figure 10c,d reveal the higher magnification of the region 2 and region 3 in Figure 10b, respectively. Both of the micrographs show a well cohered interface and bonding between SiC particle and Al matrix. A good cohesion even between the bare SiC particles and Al matrix was achieved by the FSP developed in this work, whereas other investigations reported insufficient cohesion or poor bonding at the reinforcement/matrix interface after FSP [23,24,25]. Even in the case if the alloy was not heated up to its melting temperature, such a high stability of thin copper layer at the interphase boundary between SiC and Al indicates that a kind of complete “wetting” of SiC/Al boundaries by the layer of a second solid phase was reported in other works [26,27].

**Figure 11:**
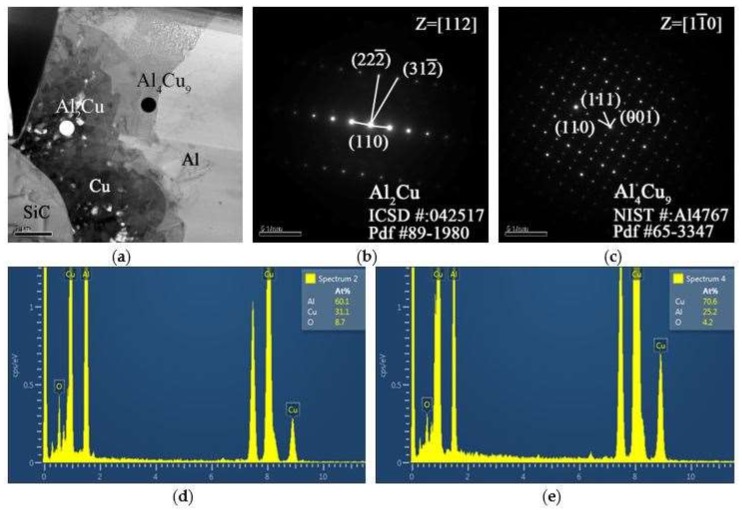
TEM micrographs, diffraction pattern and schematic index diagram with SiC/Cu reinforcement. (**a**) Bright field image, (**b**) selected area diffraction patterns (SADP) of Al_2_Cu along [112] zone axis, (**c**) SADP of Al_4_Cu_9_ along [$1\bar{1}0$], (**d**) energy dispersive spectrometer (EDS) peaks of Al_2_Cu, (**e**) EDS peaks of Al_4_Cu_9_.

The cohesion or bonding between the Cu coating and the Al matrix could be greatly affected by the formation of IMCs at the Al/Cu interface. In order to further clarify the structure of the IMCs developed at Al/Cu interface, dedicated TEM sample preparation was conduct. Samples were cut from the Al/Cu interfacial region of a Cu-coated SiC particle and were prepared by an ion mill. The TEM micrographs, the selected area diffraction patterns (SADP), and the EDS peaks are shown in Figure 11. Figure 11a depicts a transition region where SiC, Cu-coating, and the Al matrix are revealed IMC was identified using EDS. The zone axis of Al_2_Cu IMC, Z = [112] can be identified from the SADP, as shown in Figure 11b. It shows a tetragonal structure (lattice constant a = 0.487 nm, b = 0.487 nm, c = 0.607 nm). Further SADP was conducted on the inter-diffusion zone that was adjacent to the Cu coating where a second major phase Al_4_Cu_9_ was identified, as shown in Figure 11c. The zone axis of Al_4_Cu_9_ IMC is Z = [$1\bar{1}0$]. Al_4_Cu_9_ exhibits a cubic structure and the lattice constant is 0.487 nm. The EDS peaks that were associated with Al_2_Cu and Al_4_Cu_9_ are illustrated in Figure 11d,e, respectively. The SADP and EDS analyses reconfirm the IMC identification results that were obtained from EPMA.

### 3.4. Hardness Test

**Figure 12:**
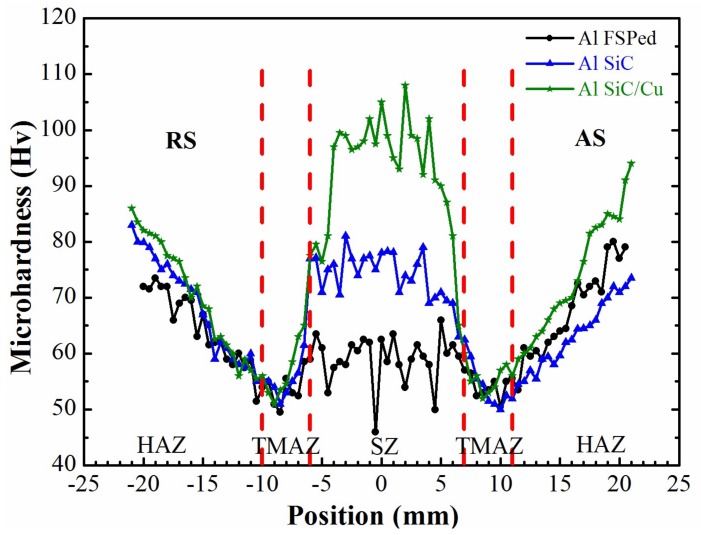
Micro-Vickers hardness distribution across the transverse section of friction stir processed Al6061 plates.

Figure 12 illustrates the hardness distribution across the transverse section of friction stir processed Al6061 plates without and with bare SiC particles or with Cu-coated particles in SZ after two-pass FSP. The SZ containing the Cu-coated SiC reinforcement exhibits the highest hardness (HV 90-HV 105), followed by the SZ containing bare SiC reinforcement (HV 70-HV80) and followed by the SZ without particulate reinforcement (HV 50-HV 63). The hardness of the base metal lies between HV 80-HV 90. The thermal mechanical affect zone (TMAZ) exhibits, in general, the lowest hardness, which is attributed to the fact that the precipitation in the Al6061-T651 were re-dissolved into matrix due to the heat that was generated during FSP [28]. The addition of both bare SiC particles and Cu-coated SiC particles could effectively improve the hardness of the SZ. The fluctuation of hardness values could be attributed to the slight unevenly distribution of particulate reinforcement across the onion ring patterns on one hand, and to the indentation on SiC particles, on the other hand. Variation of hardness values across the onion ring is due to different microstructure, grain orientation, and reinforcement particles distribution [29]. It is worthwhile to note that the hardness value in the advancing side (AS) is slightly higher than that in the retreating side (RS). During FSP, the material flows from the retreating side to the advancing side so that the advancing side exhibits a slightly elevated reinforcement particle distribution.

The improvement of hardness in the SZ containing reinforcement particles can be associated with the following mechanisms in addition to the expected particulate reinforcement. First, the SiC particles, which partake in the stirring process, may enhance the degree of plastic deformation in the stir zone and may induce additional heat during friction stir. These are beneficial to the dynamic recrystallization of the grains and may lead to further grain refinement as observed in other work [14,30]. In addition, elevated dislocation density in the microstructure of the SZ may also lead to an increase of hardness [31]. A further improvement of the hardness in the SZ by the Cu-coated SiC particles can be associated to the factors as follows:

1. The intermediate inter-diffusion layer between Cu-coating and Al matrix provides better adhesion between the reinforced SiC particle and matrix.
2. The very fine Cu debris, which is randomly distributed in the SZ provide a dispersive strengthening effect in impeding the plastic deformation.
3. The diffusion of the copper atoms into the Al matrix rendered slightly elevated Cu content, which provides an additional solid solution strengthening effect.

### 3.5. Tensile Test

Tensile test was conducted to determine the tensile properties, including ultimate tensile strength, yield strength, and percentage of elongation. Grains size, dislocation density, interface between Al matrix and reinforcement particles, and the agglomeration of reinforcement particles are the major factors that affect the tensile properties of friction stir processed specimens containing reinforcement particles [32,33]. The transverse and longitudinal tensile properties on the stir zone of AMC are listed in Table 5 and Table 6. Tensile fracture of transverse specimens always occurred in TMAZ due to deteriorated hardness and grain growth [34]. No fractures were found in the SZ, which implies a defect-free and strengthened stir zone after friction stir of particulate reinforcements into Al matrix. The transverse tensile properties are independent of the reinforcement schemes of FSP since the fracture did not occur in the SZ. Nevertheless, specimens containing reinforcement exhibited slightly higher strength and reduced elongation, as listed in Table 5. The longitudinal tensile specimens were completely taken from the stir zone. As shown in Table 6, the two-pass FSP containing Cu-coated reinforcement shows that the ultimate tensile strength and yield strength were increased by 5.5% and 8.4%, respectively. The strength of the above processes was larger than the strength of Al6061 FSP. The addition of bare SiC particles does not effectively enhance the tensile strength. A two-pass stir could generally improve the tensile strength of the SZ, which is attributed to grain refinement after recrystallization and the improved dispersion of fine Cu particles and Cu atoms in the matrix [35]. With the improved homogeneity of reinforcement particles distribution and the improved cohesion between reinforcing particle and Al matrix, the increase in ultimate tensile strength (UTS) and yield strength (YS) can be understood. Moreover, the better bonding between the reinforcing particles and the Al matrix may be partly attributed to Orowan strengthening mechanism [36]. The elongation of material was obviously enhanced due to the grain refinement in the stir zone after three schemes of FSP. The test result of one-pass and two-pass FSP shows that the elongation of two-pass was greater than that of one-pass due to the improved dispersion of fine particles. Due to the outstanding stability of FSP, the SiC particles with different surface-coating conditions could be homogeneously dispersed in the matrix. Therefore, the mechanical properties of the stir zone would be greatly improved.

### 3.6. Fractography on Tensile Specimens

The contrast of the backscattered electron image depends on the atomic number (Z) of the sample material. Materials with elements that were composed of a higher atomic number yield more backscattered electrons than lower Z elements. The atomic number of Al, Cu, Si, and C are 13, 29, 14, and 6, respectively, so Cu shows the brightest in back-scatter detector (BSE) image. However, the atomic number of Al and Si are very close so it is hard to distinguish in BSE image. Therefore, EDS analysis is adopted to identify the element.

**Figure 13:**
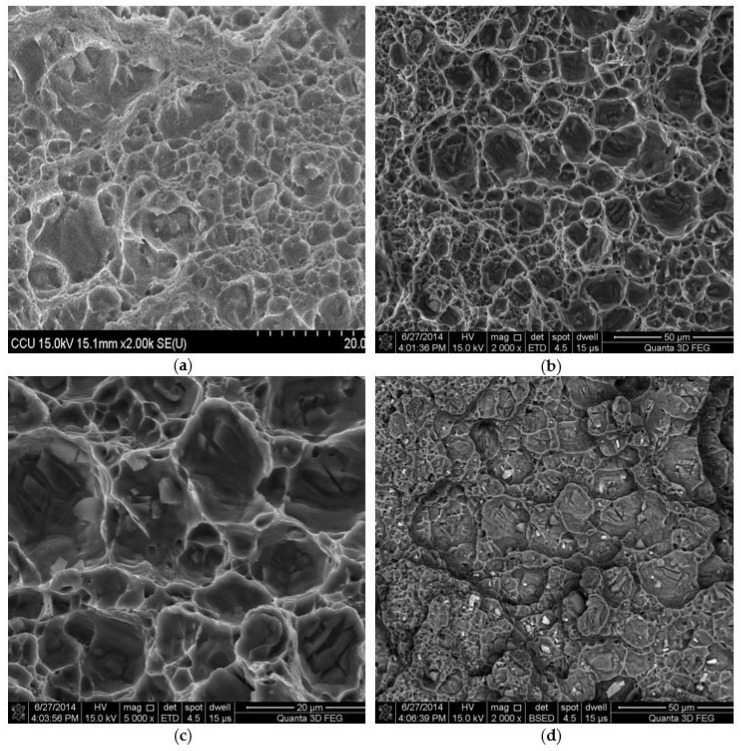
SEM fractographs of tensile fracture surfaces. (**a**) SZ containing bare SiC, (**b**) SZ containing Cu-coated SiC, (**c**) higher magnification at 5000× of (**b**), and (**d**) Base-scatter detector (BSE) image of SZ containing Cu-coated SiC.

Figure 13a shows the fracture surface of SZ containing bare SiC reinforcement. It generally exhibits the feature of ductile fracture. Figure 13b,c depicts the ductile fracture surface of SZ containing the Cu-coated SiC reinforcement. A few SiC particles that were embedded in the dimple fracture can be observed. Figure 13d shows the BSE image that reveals a uniform distributed of Cu-coated SiC reinforced particles and very good cohesion between Cu-coated SiC particles and the Al matrix.

## 4. Conclusions

Locally particulate reinforced AMC was fabricated by stirring SiC and Cu-coated SiC particulate reinforcement into Al6061-T651 matrix using friction stir processing. Based on the present results, the following conclusions are made:

1. The microstructure reveals a defect-free stir zone characterized by uniform distribution of SiC or Cu-coated SiC particulate reinforcement. Perfect cohesion between SiC particles and Al-matrix or between Cu-coated SiC particles and Al matrix has been achieved.
2. During friction stir processing, the intermetallic compounds, such as Al_2_Cu and Al_4_Cu_9_, were in-situ formed at the inter-diffusion layer between the Cu layer around the SiC particles and the Al matrix. The formation of the IMCs may enhance the cohesion of the reinforced particles to the matrix and results in the effective improvement in micro-hardness and tensile strength.
3. Peeling-off Cu-coating from the SiC particles during FSP could result in the dispersion of very fine Cu debris in the matrix and provide further dispersion strengthening and solid solution strengthening of Al6061.
4. The concept that is realized in this work may open a new way of fabrication of particulate reinforced metal matrix composites.

## Figures and Tables

**Table 1:** Chemical compositions (wt %) of Al6061-T651 sheet.

Elements	Si	Fe	Cu	Mn	Mg	Cr	Al
Weight %	0.478	0.800	0.284	0.148	0.968	0.277	Balance

**Table 2:** Mechanical properties of Al6061-T651.

Base Material	UTS(MPa)	YS(MPa)	Elongation (%)
Al6061-T651	312	289	15.5

**Table 3:** Chemical compositions of the electroless copper plating solution.

Components	Concentration
CuSO_4_·5H_2_O	0.03 mol/L
EDTA	0.1 mol/L
2,2′-bipyridyl	5 × 10^−4^ mol/L
Potassium Ferrocyanide	5 × 10^−4^ mol/L
Formaldehyde (35 wt %)	13.5 mL/L
SiC particles	2 g/L

**Table 4:** Chemical compositions of the etching solution.

HF	HCl	HNO_3_	DI Water
12 mL	12 mL	12 mL	150 mL

**Table 5:** Transverse tensile properties of specimens after different schemes of FSP.

Reinforcement Schemes of SZ	UTS (MPa)	$\sigma_{y}$ (MPa)	$\varepsilon_{f}$ (%)	Fracture Location
Al FSPed 1pass	195	134	10.2	TMAZ
Al-SiC 1pass	199	134	8.9	TMAZ
Al-SiC/Cu 1pass	205	137	9.2	TMAZ
Al FSPed 2pass	192	123	10.7	TMAZ
Al-SiC 2pass	197	130	10.4	TMAZ
Al-SiC/Cu 2pass	243	175	9.6	TMAZ

**Table 6:** Longitudinal tensile properties of specimens after different schemes of FSP.

Reinforcement Schemes of SZ	UTS (MPa)	$\sigma_{y}$ (MPa)	$\varepsilon_{f}$ (%)
Al6061-T651	312	289	15.5
Al FSPed 1pass	246	128	35.5
Al-SiC 1pass	222	122	14.2
Al-SiC/Cu 1pass	235	126	18.4
Al FSPed 2pass	251	130	37.0
Al-SiC 2pass	240	125	24.7
Al-SiC/Cu 2pass	265	141	20.1
